# Enterovirus 2A^pro^ Cleavage of the YTHDF m^6^A Readers Implicates YTHDF3 as a Mediator of Type I Interferon-Driven JAK/STAT Signaling

**DOI:** 10.1128/mBio.00116-21

**Published:** 2021-04-13

**Authors:** Jonathan P. Kastan, Martine W. Tremblay, Michael C. Brown, Joseph D. Trimarco, Elena Y. Dobrikova, Mikhail I. Dobrikov, Matthias Gromeier

**Affiliations:** a Department of Neurosurgery, Duke University Medical Center, Durham, North Carolina, USA; b University Program in Genetics and Genomics, Duke University Medical Center, Durham, North Carolina, USA; c Molecular Genetics and Microbiology, Duke University Medical Center, Durham, North Carolina, USA; University of Colorado School of Medicine

**Keywords:** 2A protease, enterovirus, Jak/Stat signaling, poliovirus, YTHDF proteins, innate immunity, interferon stimulated gene, m^6^A modification, type I interferon, type III interferon

## Abstract

Enteroviruses (EV) deploy two proteases that mediate viral polyprotein cleavage and host cell manipulation. Here, we report that EV 2A proteases cleave all three members of the YTHDF protein family, cytosolic *N*^6^-methyladenosine (m^6^A) “readers” that regulate target mRNA fate. YTHDF protein cleavage occurs very early during infection, before viral translation is detected or cytopathogenic effects are observed. Preemptive YTHDF protein depletion enhanced viral translation and replication but only in cells with restrained viral translation, signs of inefficient 2A protease activity, and protective innate host immune responses. This effect corresponded with repression of interferon (IFN)-stimulated gene (ISG) induction, while type I/III IFN production was not significantly altered. Moreover, YTHDF3 depletion impaired JAK/STAT signaling in cells treated with type I, but not type II, IFN. YTHDF3 depletion’s stimulatory effect on viral dynamics was dampened by JAK/STAT blockade and enhanced by type I IFN pretreatment of cells. We propose that EV 2A proteases cleave YTHDF proteins to antagonize ISG induction in infected cells.

## Abstract (precis)

It is believed that ∼7,000 messenger RNAs (mRNAs) are subject to *N*^6^-methyladenosine modification. The biological significance of this remains mysterious.

## INTRODUCTION

*N*^6^-methyladenosine (m^6^A) modification of mRNAs influences diverse processes governing RNA metabolism, including splicing, nucleocytoplasmic transport, template stability, and translation (1–9). The YT521-B homology domain-containing proteins (YTHDF1 to 3), labeled “m^6^A readers” due to their affinity for m^6^A-modified RNA (10, 11), assume key roles in these processes that remain poorly understood.

Positive-sense single-strand (+ss) RNA viruses are a central focus for uncovering the biological significance of YTHDFs, because their genomes are m^6^A-modified (12, 13) and because m^6^A is implicated in controlling antiviral type I IFN (14, 15) and IFN-stimulated gene (ISG) responses (2, 3). If YTHDFs are involved in orchestrating innate antiviral immunity, they may be targets of viral countermeasures.

+ssRNA viruses deploy viral proteases for processing of their polyproteins. In some instances, these proteases also execute targeted cleavage of host cell proteins as part of the viral replication strategy (16). Enteroviruses (EV) encode two cysteine proteases; 3C^pro^ processes most polyprotein cleavages. Meanwhile, 2A^pro^ mainly cleaves the polyprotein at the P1-P2 junction (17) but degrades host proteins with vital functions in translation (eukaryotic initiation factor [eIF] 4G1/2) and nucleocytoplasmic transport (nuclear pore complex components Nup153 and p62) (18, 19).

An unbiased screen for human host cell targets of 2A^pro^ yielded a very small set of proteins containing the conserved consensus poliovirus (PV) 2A^pro^ auto-proteolytic cleavage motif (LTTY′G) (17). Among them were YTHDF1 and YTHDF3. Here, we show that YTHDF1, 2, and 3 are cleaved in EV-infected HeLa cells at a rate similar to that of eIF4G1, the emblematic host cell target for 2A^pro^ (18). YTHDF3 depletion enhanced viral translation and replication exclusively in cell lines that mount robust, protective innate antiviral responses to infection. Functional studies with YTHDF3 depletion in EV-infected cells revealed elevated induction of IFN response factor 3 (IRF3) phosphorylation and IFN-β/λ1 mRNAs, while IFN-β/λ1 release was not significantly altered. However, IFN-stimulated gene (ISG) induction was diminished substantially. The stimulatory effects of YTHDF3 knockdown on EV dynamics were dampened by blockade of the JAK/STAT pathway and enhanced by IFN pretreatment in cells. Indeed, our findings implicate 2A^pro^-mediated YTHDF3 cleavage as a potential viral strategy to impair JAK/STAT signaling in infected host cells.

## RESULTS

### Enterovirus 2A^pro^ mediates cleavage of YTHDF proteins.

**FIG 1:**
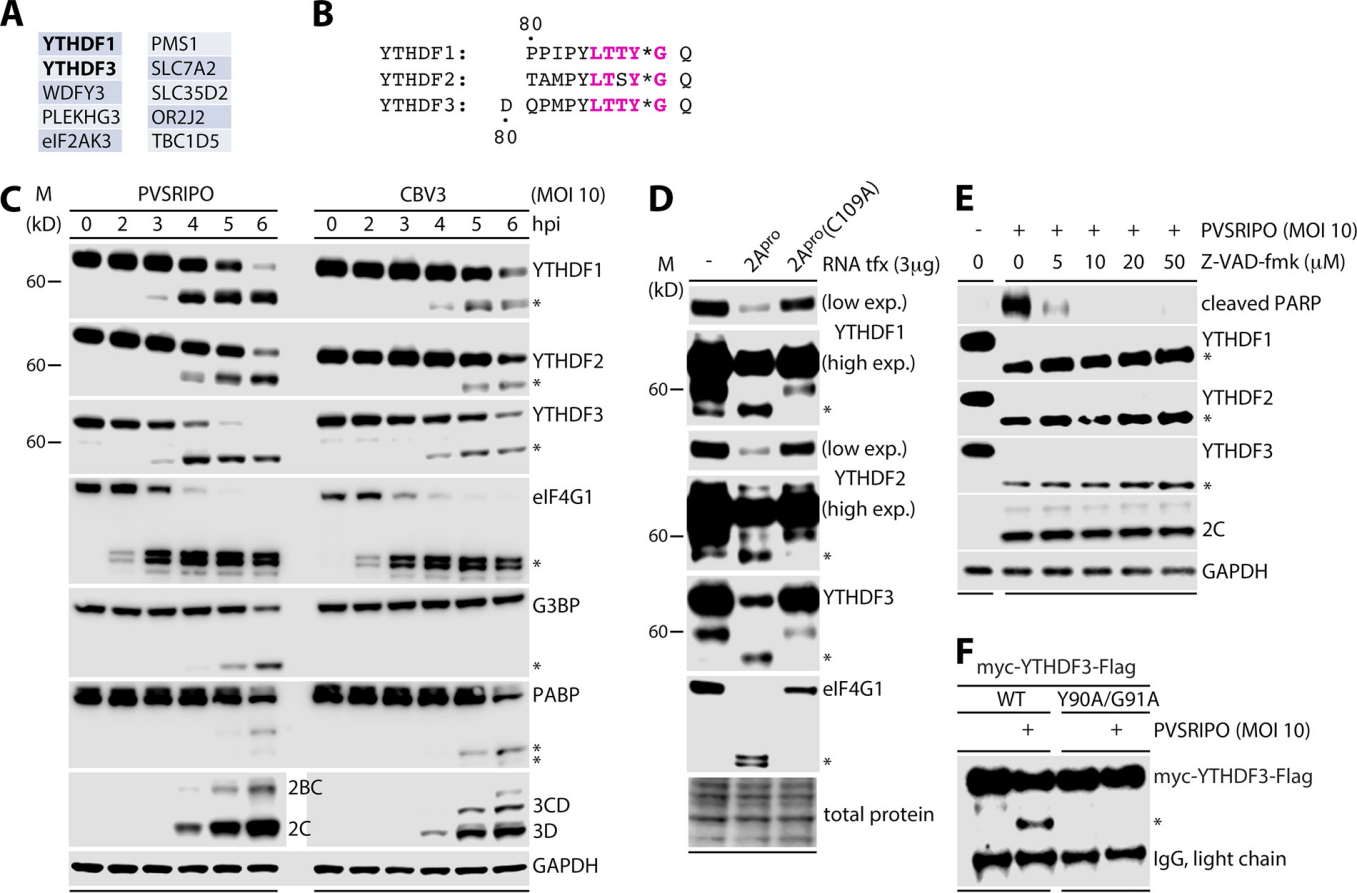
Enterovirus 2A^pro^ executes early cleavage of YTHDF1 to 3 in infected host cells. (A) Human proteins that contain the PV 2A^pro^ LTTY′G motif as determined by BLAST search. (B) Location and conservation of putative PV 2A^pro^ cleavage sites (*) in YTHDF1 to 3. See Fig. S1 for schematic representation of the YTHDF1 to 3 cleavage fragments. (C) HeLa cells were infected with PVSRIPO or coxsackievirus B3 (CBV3) (MOI 10) and lysed at the indicated hours postinfection (hpi) for immunoblot analysis as shown; *denotes cleavage fragments for each target. (D) HeLa cells were transiently transfected with *in vitro* transcribed RNA (3 μg) encoding either WT PV 2A^pro^ or the catalytically inactive C109A 2A^pro^ variant (28) and lysed for immunoblot analysis 12 h posttransfection. (E) HeLa cells were infected with PVSRIPO (MOI 10; 8 h) in the presence of increasing concentrations of Z-VAD-fmk and lysed for immunoblot analysis. (F) HeLa cells were transfected with myc-YTHDF3-Flag (WT) or myc-YTHDF3(Y90A/G91A)-Flag for 24 h prior to PVSRIPO infection (MOI 10; 8 h). Samples were then subjected to RIPA lysis, IP with anti-Flag beads, and anti-Flag immunoblot.

To establish a list of candidate host proteins that may be targeted by PV 2A^pro^ for cleavage, we performed a proteome-wide BLAST search for the Leu-Thr-Thr-Tyr-Gly (LTTY′G) pentamer, the conserved 2A^pro^-targeted sequence context at the cleavage site separating the P1 and P2 precursor polypeptides of PV (17). A 5-amino acid (aa) motif was chosen, as this is the consensus context reported to be critical for 2A^pro^ substrate recognition (20). This approach yielded a list of only 10 putative 2A^pro^ targets with an exact match for this pentamer (Fig. 1A). Notably, the list included YTHDF1 and 3, RNA-binding proteins with preference for methylated RNA (10, 11). YTHDF2 was not picked up in our screen, because it contains an LTSY′G pentamer (Fig. 1B). Ser in position P2 of the 2A^pro^ target sequence does not diminish proteolytic processing (20); therefore, YTHDF2 also is a plausible 2A^pro^ target. Due to their implication in posttranscriptional regulation of viral genomic RNAs (21), in the life cycle of RNA viruses (13, 22), and in host innate antiviral immunity (15, 23), we decided to investigate YTHDF1 to 3 as putative 2A^pro^ substrates and codeterminants of the EV-host cell relationship.

10.1128/mBio.00116-21.2 FIG S1 Location of the PV 2A^pro^ consensus cleavage motifs in YTHDF1 to 3 and predicted approximate sizes of cleavage fragments. Cleavage of YTHDF1 to 3 by enterovirus 2A^pro^ is predicted to yield N- and C-terminal fragments of ∼10-kDa and ∼50-kDa size, respectively. Download FIG S1, TIF file, 0.1 MB. Copyright © 2021 Kastan et al. 2021 Kastan et al. https://creativecommons.org/licenses/by/4.0/ This content is distributed under the terms of the Creative Commons Attribution 4.0 International license.

To elucidate the dynamics of YTHDF protein expression/cleavage during EV infection, we infected HeLa cells with an attenuated poliovirus (24) containing a heterologous internal ribosomal entry site (IRES) of human rhinovirus type 2 (PVSRIPO) (25) or a related EV, coxsackievirus B3 (CBV3; Fig. 1C). This revealed that YTHDF1, 2, and 3 are cleaved over the course of infection, with rapid loss of full-length protein and the appearance of an ∼50- to 55-kD fragment corresponding to the predicted size of the C-terminal fragment produced by cleavage at LT^T^/_S_Y′G (Fig. S1). The earliest cleavage of YTHDF1 and 3 was observed at 3 to 4 hpi in PVSRIPO-infected cells, prior to detection of viral translation products, with comparable kinetics to those of the signature 2A^pro^-mediated cleavage of eIF4G1 (Fig. 1C). In contrast, earlier reported cleavages attributed to the poliovirus 3C protease, Ras-GAP SH3 domain-binding protein (G3BP) (26), and poly(A) binding protein (PABP) (27) did not occur before 5 hpi in PVSRIPO/CVB3 infected cells, an interval with rampant viral translation (Fig. 1C). To determine if 2A^pro^ was sufficient for cleavage of YTHDF1 to 3, as suggested by the presence of the PV 2A^pro^ autocatalytic cleavage motif (Fig. 1A and B), we transfected *in vitro* transcribed RNA encoding wild type (WT) poliovirus 2A^pro^ or the catalytically inactive 2A^pro^(C109A) (28) (Fig. 1D). WT 2A^pro^ expression yielded YTHDF1 to 3 cleavage, while the catalytically inactive variant did not (Fig. 1D).

PV/CBV3 infection of HeLa cells produces profound cytotoxicity associated with generalized, indiscriminate proteolytic degradation of the host proteome at the peak of unfettered viral protein synthesis and the onset of morphologically evident cytopathogenic effects (CPE). Therefore, it is pivotal to distinguish early proteolytic events that occur prior to the onset of CPE, and that shape the virus-host relationship, from late, wholesale lytic degradation. To eliminate a potential role of organized cell death programs in the cleavage of YTHDF1 to 3 in EV-infected cells, we carried out infections in cells cotreated with escalating concentrations of Z-VAD-fmk, a pan-caspase inhibitor (Fig. 1E). This reduced cleavage of PARP, a caspase substrate, even at the lowest concentration tested but had no effect on YTHDF1 to 3 cleavage at any concentration (Fig. 1E).

Lastly, we transfected cells with constructs expressing WT myc-YTHDF3-Flag or a variant with the Tyr-Gly (Y′G) cleavage site mutated to Ala-Ala (Fig. 1F). These cells were then infected with PVSRIPO, lysed at 8 hpi, and processed for Flag immunoprecipitation (Fig. 1F). This revealed the characteristic ∼54-kD cleavage fragment in cells expressing the WT YTHDF3 construct but not the variant with the A′A substitution of the 2A^pro^ cleavage site. In aggregate, our findings indicate that YTHDF1 to 3 are cleaved by EV 2A^pro^ at predicted consensus 2A^pro^ cleavage sites early during infection, prior to the onset of bulk viral translation and host CPE. This scenario suggests that viral cleavage of YTHDF1 to 3 may be a critical factor in the EV-host relationship.

### YTHDF3 antagonizes PVSRIPO replication and translation.

**FIG 2:**
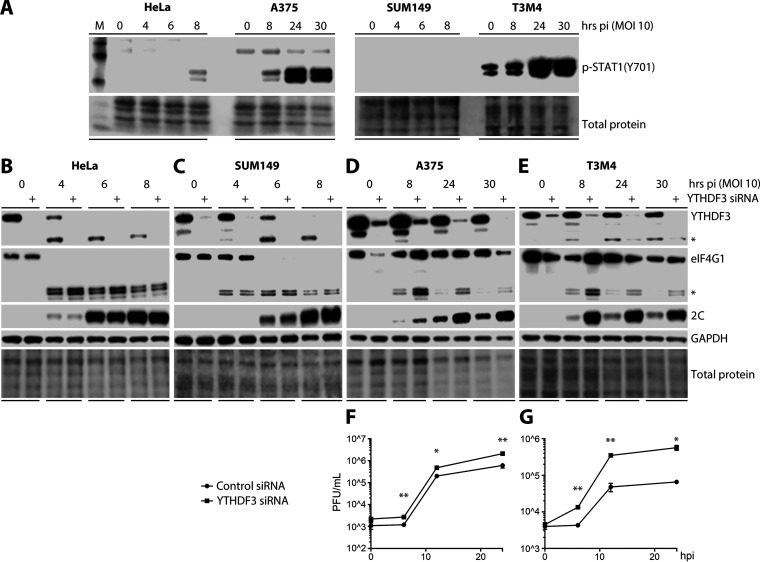
Preemptive YTHDF3 depletion stimulates PVSRIPO replication in cells with effective innate antiviral defenses. (A) HeLa, SUM149, A375, and T3M4 cells were infected with PVSRIPO (MOI 10) for the indicated times prior to lysis for immunoblot analysis of p-STAT1(Y701) induction. See Fig. S2 for cytopathogenicity analyses. (B) HeLa, (C) SUM149, (D) A375, or (E) T3M4 cells were transfected with ctrl siRNA or siRNA targeting YTHDF3 48 h prior to PVSRIPO infection (MOI 10) and lysis at the indicated intervals for immunoblot analysis. See Fig. S3 and S4 for extended analyses. (F) A375 or (G) T3M4 cells were transfected with ctrl siRNA or siRNA targeting YTHDF3 48 h prior to PVSRIPO infection (MOI 10) for one-step growth curve analysis (see Materials and Methods). Plaque assays were performed to determine viral titers/sample (PFU/ml) at the indicated intervals (*n* = 3). Graphs represent mean ± SEM; * and ** correspond to *P* < 0.05 and *P* < 0.005, respectively.

To decipher the physiologic significance of viral YTHDF1 to 3 cleavage for host-virus interactions, we focused on YTHDF3, as it was most rapidly cleaved during EV infection. Because innate antiviral immunity is a central area of interest for deciphering m^6^A and YTHDF biology, we employed a panel of four cell lines with diverse permissiveness for viral, m^7^G-cap-independent translation of PVSRIPO and, accordingly, distinct innate antiviral host responses to infection (Fig. 2A). PVSRIPO elicits robust type I/III IFN responses, orchestrated by host sensing of viral RNA signatures, engaging the pattern recognition receptor (PRR) melanoma differentiation associated protein 5 (MDA5) (29, 30). This is evident, as phosphorylation of STAT1(Y701), downstream of the type I/III IFN receptors, was induced upon PVSRIPO infection (Fig. 2A).

10.1128/mBio.00116-21.3 FIG S2 Distinct levels of cytopathogenicity in response to PVSRIPO infection of HeLa, Sum149, A375, and T3M4 cells. HeLa, Sum149, A375, and T3M4 cells were infected with PVSRIPO (MOI 10) for the indicated times when photomicrographs were taken to assess the extent of cytopathogenic effects. Download FIG S2, TIF file, 2.8 MB. Copyright © 2021 Kastan et al. 2021 Kastan et al. https://creativecommons.org/licenses/by/4.0/ This content is distributed under the terms of the Creative Commons Attribution 4.0 International license.

10.1128/mBio.00116-21.4 FIG S3 YTHDF3 depletion with distinct siRNA probes substantially enhances viral translation and eIF4G1 cleavage at MOIs of 10, 1, or 0.1 in infected A375 cells. A375 cells were transfected with ctrl siRNA or one of two siRNAs targeting YTHDF3 (see Table S1) 48 h prior to PVSRIPO infection at the indicated MOIs and lysis at 30 hpi for immunoblot analysis. Download FIG S3, TIF file, 0.5 MB. Copyright © 2021 Kastan et al. 2021 Kastan et al. https://creativecommons.org/licenses/by/4.0/ This content is distributed under the terms of the Creative Commons Attribution 4.0 International license.

10.1128/mBio.00116-21.1 TABLE S1 Sequences of RT-qPCR primers and siRNAs used in this study. Download Table S1, TIF file, 1.8 MB. Copyright © 2021 Kastan et al. 2021 Kastan et al. https://creativecommons.org/licenses/by/4.0/ This content is distributed under the terms of the Creative Commons Attribution 4.0 International license.

10.1128/mBio.00116-21.5 FIG S4 Depletion of YTHDF1 and YTHDF2 enhances PVSRIPO translation at MOIs of 10, 1, or 0.1 in infected A375 cells. A375 cells were transfected with ctrl siRNA or siRNA targeting YTHDF1 or YTHDF2 (see Table S1) 48 h prior to PVSRIPO infection at the indicated MOIs and lysis at 30 hpi for immunoblot analysis. *Increased viral translation upon YTHDF2 depletion in PVSRIPO (MOI 10, 1)-infected A375 cells produced substantial cytotoxicity, which broadly reduced total protein. This led to reduced levels of YTHDF1 and the GAPDH loading control in the corresponding samples. Download FIG S4, TIF file, 0.5 MB. Copyright © 2021 Kastan et al. 2021 Kastan et al. https://creativecommons.org/licenses/by/4.0/ This content is distributed under the terms of the Creative Commons Attribution 4.0 International license.

HeLa (cervical carcinoma, human papillomavirus 18 infected) and SUM149 (breast cancer) cells permit rampant early viral translation and efficient eIF4G1 cleavage (substantial reduction of intact eIF4G1 prior to 6 hpi) and do not mount effective antiviral host responses to PVSRIPO (multiplicity of infection [MOI] 10). This is evident by a failure to properly induce p-STAT1(Y701) upon PVSRIPO infection (Fig. 2A). SUM149 did not respond with p-STAT1(Y701) induction; delicate p-STAT1(Y701) immunoblot signal in PVSRIPO-infected HeLa cells occurred only at 8 hpi, at an interval with robust viral translation and after the onset of CPE (Fig. 2A). Accordingly, there was an inability to protect cells from rapid eIF4G1 cleavage (Fig. 2B), the early burst of viral translation (Fig. 2B), and early (12 hpi) virus-induced death (Fig. S2). Thus, infection of HeLa and SUM149 cells resulted in overt CPE by 12 hpi (Fig. S2). Preemptive YTHDF3 depletion in HeLa and SUM149 cells had no discernible effect on PVSRIPO translation or eIF4G1 cleavage (Fig. 2B and C). Thus, in cells that permit unimpeded early viral translation and 2A^pro^ activity yielding efficient eIF4G1 and YTHDF protein cleavage, YTHDF3 depletion did not change the host-PVSRIPO relationship (Fig. 2B and C).

Compared to that in HeLa and SUM149 cells, PVSRIPO translation in A375 (melanoma) and T3M4 (pancreatic ductal adenocarcinoma) cells was delayed and eIF4G1 cleavage was inefficient (Fig. 2D and E). Indeed, PVSRIPO-infected A375 and T3M4 cells retained substantial amounts of intact eIF4G1 at 24 hpi, while HeLa and SUM149 cells were already lysed at that interval (Fig. 2B and C; Fig. S2). Accordingly, A375 and T3M4 cells responded with vigorous innate immune responses to PVSRIPO (MOI 10) infection, evident by abundant p-STAT1(Y701) induction (Fig. 2A). Corresponding with a protective host innate antiviral response delaying the early burst of viral translation and obstructing eIF4G1 cleavage, overt CPE was delayed/incomplete in A375 cells and absent in T3M4 cells (Fig. S2). A375 cells had no baseline p-STAT1(Y701) and responded with p-STAT1(Y701) induction only after PVSRIPO infection (Fig. 2A). In contrast, T3M4 exhibited baseline p-STAT1(Y701) signaling, indicating intrinsically active JAK/STAT1 signaling in the absence of virus challenge (Fig. 2A).

YTHDF3 depletion profoundly affected viral translation and eIF4G1 cleavage in PVSRIPO-infected (MOI 10) A375 and T3M4 cells (Fig. 2D and E). Furthermore, PVSRIPO propagation, which is surprisingly robust in the presence of an active, protective innate antiviral response (31), was enhanced by YTHDF3 depletion in accordance with the level of induction of viral translation (Fig. 2D to G). In T3M4 cells, YTHDF3 depletion propelled PVSRIPO propagation ∼9-fold at 24 hpi (Fig. 2F). Divergent values at the 0 hpi interval in Fig. 2F were due to inherent variability of virus recovery from crude cell lysates in the assay; YTHDF3 depletion had no significant effect on virus attachment or entry (Fig. 2G). The stimulatory effects of YTHDF3 depletion on viral translation and eIF4G1 cleavage (Fig. 2D) were confirmed with a distinct siRNA probe and in A375 cells infected at a range of MOIs of 10, 1, and 0.1 (Fig. S3). We also assessed the effects of YTHDF1 and 2 knockdown in A375 cells infected with PVSRIPO at MOIs of 10, 1, and 0.1 (Fig. S4). Depletion of YTHDF1 or 2 had comparable stimulatory effects on viral translation and eIF4G1 cleavage to those of YTHDF3 knockdown in A375 cells (Fig. 2D; Fig. S3 and S4). Together, these observations point toward a role for YTHDF1 to 3 in shaping the PVSRIPO-host relationship through their influence on the innate antiviral defense.

### YTHDF3 facilitates signaling downstream of IFN production induced by EV infection.

**FIG 3:**
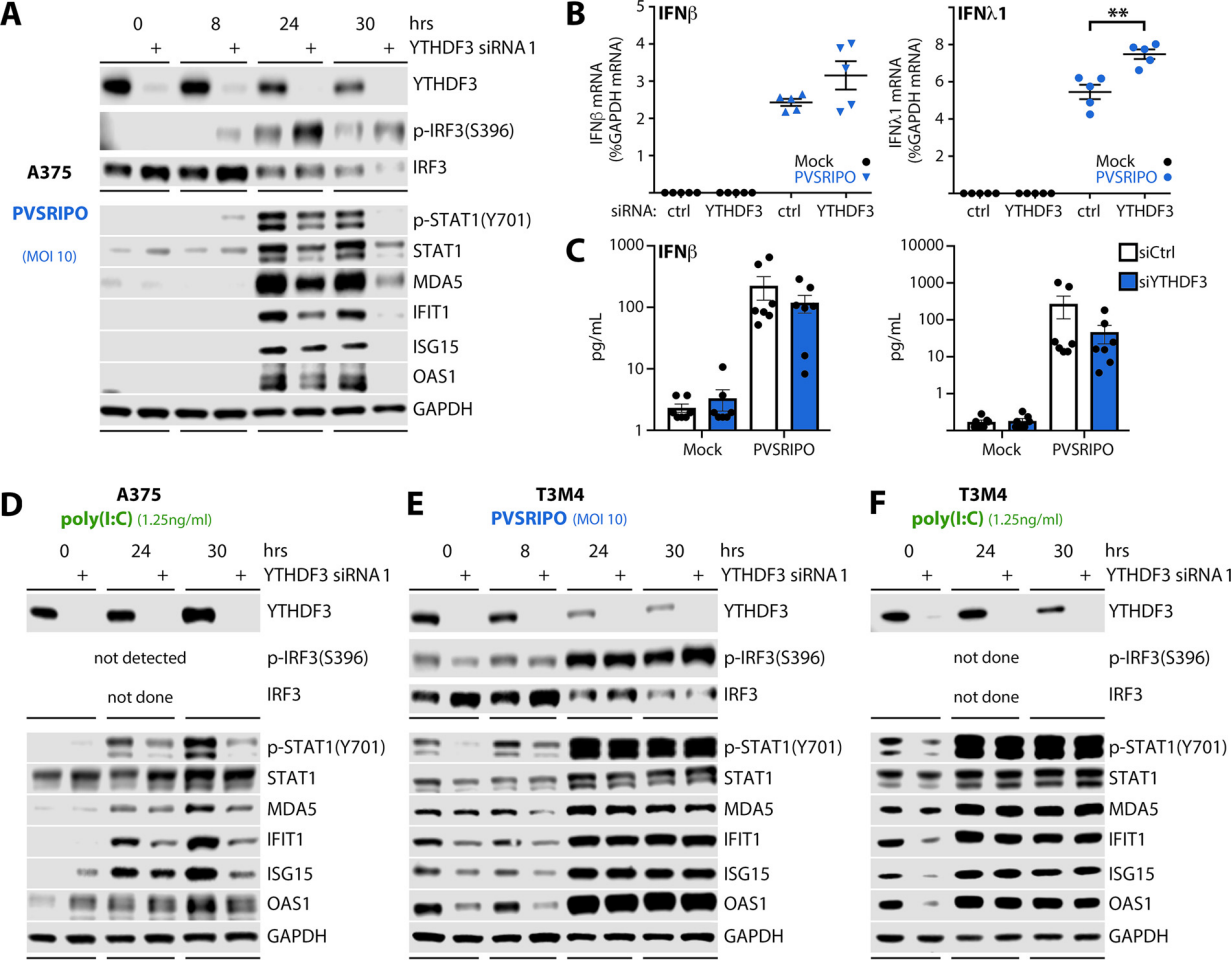
YTHDF3 depletion inhibits the type I/III IFN-driven ISG response. (A) A375 or (E) T3M4 cells were transfected with ctrl siRNA or siRNA targeting YTHDF3 48 h prior to PVSRIPO infection (MOI 10) and lysis at the indicated time point for immunoblot analysis. (B and C) A375 cells were transfected with ctrl siRNA or siRNA targeting YTHDF3 48 h prior to PVSRIPO infection (MOI 10, 30 h). (B) Cells were lysed for qRT-PCR analysis of the indicated transcripts (*n* = 5). (C) Supernatants were analyzed for type I/III IFN secretion by multiplex cytokine analysis (*n* = 7). (D) A375 or (F) T3M4 cells were transfected with ctrl siRNA or siRNA targeting YTHDF3 48 h prior to poly(I:C) transfection (1.25 ng/ml) and lysis at the indicated time point for immunoblot analysis. Graphs represent mean ± SEM; *, **, and *** correspond to *P* < 0.05, *P* < 0.005, and *P* < 0.0005, respectively.

To determine if YTHDF3 is a factor involved in the innate antiviral response, we depleted YTHDF3 in A375 and T3M4 cells prior to infection with PVSRIPO (MOI 10) or transfection with high molecular weight (HMW) poly(I:C) and examined activation of antiviral signaling (Fig. 3). It has been shown that transfection with HMW poly(I:C) mimics innate activation via Toll-like receptor 3 and MDA5 (30, 32). Our approach tested four stages of the innate response to PVSRIPO/poly(I:C) in A375 cells: (i) IRF3(S396) phosphorylation, an immediate consequence of TBK1:IKKε activation after engaging MDA5 (Fig. 3A), (ii) induction of type I/III IFN mRNAs upon IRF3 activation (Fig. 3B), (iii) production and release of type I/III IFN (Fig. 3C), and (iv) induction of p-STAT1(Y701) and ISGs (STAT1, MDA5, IFIT1, ISG15, OAS1) downstream of the type I/III IFN receptors (Fig. 3A and D).

A375 cells responded to PVSRIPO infection with induction of IRF3(S396) phosphorylation as early as 8 hpi (Fig. 3A). This event was enhanced upon YTHDF3 depletion throughout the time course at 8, 24, and 30 hpi (Fig. 3A). A global loss of immunoblot signal at 30 hpi in cells treated with YTHDF3 siRNA (also evident in Fig. 2D) is due to enhanced viral translation/propagation resulting in accentuated CPE upon YTHDF3 depletion. Elevated levels of IRF3 activation corresponded with significantly enhanced induction of type III (λ1) IFN transcripts at 30 hpi, while induction of type I (β) IFN mRNA was also increased, albeit insignificantly (Fig. 3B). Meanwhile, YTHDF3 depletion diminished PVSRIPO-induced IFN-β/λ1 release; however, this effect was not statistically significant (Fig. 3C). Opposite effects of YTHDF3 depletion on type I/III IFN template abundance versus those on type I/III IFN protein levels may be related to increased virus-induced cleavage of eIF4G and broad suppression of host protein synthesis that this event portends (Fig. 2D and E). This may impinge on type I/III IFN biosynthesis. Overall, these findings indicate that YTHDF3 is not involved in sensing viral RNA signatures or in the signaling pathway culminating in type I/III IFN release orchestrated by MDA5.

The principal effect of YTHDF3 depletion on the innate host response to PVSRIPO infection was dampened induction of ISGs (STAT1, MDA5, IFIT1, ISG15, OAS1) (Fig. 3A). This was also observed in YTHDF3-depleted cells transfected with poly(I:C) (Fig. 3D). A375 cells did not respond with detectable IRF3(S396) phosphorylation to poly(I:C) transfection (Fig. 3D).

In T3M4 cells—which exhibit baseline IRF3(S396) phosphorylation and ISG expression in the absence of PVSRIPO infection/poly(I:C) transfection—the response to YTHDF3 depletion was broadly reduced p-STAT1(Y701) and ISG expression at baseline (0 hpi) and at 8 hpi (Fig. 3E). This effect was overcome by innate “super”-activation upon PVSRIPO (MOI 10) infection at 24 and 30 hpi, which elevated the innate host antiviral response beyond baseline levels (Fig. 3E). As with A375 cells, poly(I:C) transfection recapitulated the effects of YTHDF3 depletion on the ISG response induced by PVSRIPO in T3M4 cells (Fig. 3F).

Our findings indicate that YTHDF3 depletion stimulates PVSRIPO translation and replication through interfering with the innate host response at a node that occurs after type I/III IFN release. In A375 cells, YTHDF3 depletion inhibited ISG induction that was triggered by PVSRIPO infection or poly(I:C) transfection. In T3M4 cells, with active baseline STAT1 signaling, YTHDF3 depletion diminished innate activation at 0 to 8 hpi, during the critical early phase of PVSRIPO infection. The latter may explain the more robust stimulatory effect on viral translation upon YTHDF3 depletion in T3M4 cells compared to that in A375 cells (Fig. 2D and E).

### YTHDF3 facilitates JAK/STAT1 signaling downstream of type I IFN.

**FIG 4:**
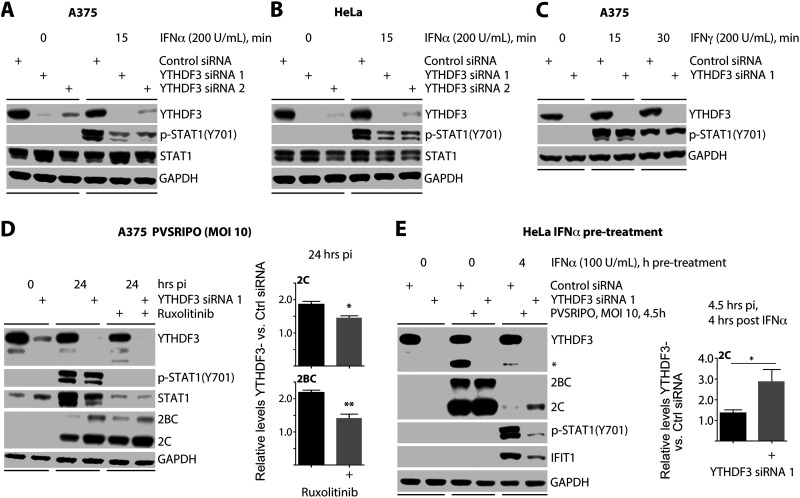
YTHDF3 depletion inhibits type I IFN-driven JAK/STAT1 signaling. (A) A375 or (B) HeLa cells were transfected with ctrl siRNA or one of two siRNAs targeting YTHDF3 48 h prior to treatment with 200 U/ml of IFN-α. (C) A375 cells were transfected with ctrl siRNA or siRNA targeting YTHDF3 48 h prior to treatment with 200 U/ml of IFN-γ and lysed at the indicated time point for immunoblot analysis. (D) A375 cells were transfected with ctrl siRNA or siRNA targeting YTHDF3 48 h prior to PVSRIPO infection (MOI 10; 24 h) in the presence or absence of Ruxolitinib (50 nM), and the relative induction of viral protein expression upon YTHDF3 depletion was quantified for the two conditions. (E) HeLa cells were transfected with ctrl siRNA or siRNA targeting YTHDF3 48 h prior to PVSRIPO infection (MOI 10; 4.5 h), with or without 4 h pretreatment with 100 U/ml of IFN-α, and the relative induction of viral protein expression upon YTHDF3 depletion was quantified for the two conditions (*n* = 5). Graphs represent mean ± SEM; *, **, and *** correspond to *P* < 0.05, *P* < 0.005, and *P* < 0.0005, respectively.

Our data suggest that YTHDF3 depletion affects the host cell response to PVSRIPO infection at a step between type I/III IFN release and p-STAT1(Y701) induction, which points to a defect in IFN-dependent JAK/STAT1 pathway activation. To test this, we examined the effect of YTHDF3 depletion on A375 cells treated with either (type I) IFN-α or (type II) IFN-γ, which signal to and converge on STAT1 through diverse receptor signaling modules (Fig. 4A to C). YTHDF3 depletion reduced p-STAT1(Y701) induction upon treatment with IFN-α in A375 (Fig. 4A) and HeLa cells (Fig. 4B) but not after treatment with IFN-γ (Fig. 4C).

Our data show that YTHDF3 positively regulates type I IFN-induced JAK/STAT1 activity. To confirm this, we depleted YTHDF3 in A375 cells, in the presence or absence of Ruxolitinib (Rux), a specific JAK1/2 inhibitor (Fig. 4D). If YTHDF3 influences the PVSRIPO-host innate immune relationship by facilitating JAK/STAT1 signaling, Rux should mitigate the effects of YTHDF3 depletion on PVSRIPO described in Fig. 2D. This was indeed the case, as Rux significantly dampened the stimulatory effect of YTHDF3 depletion on PVSRIPO translation (Fig. 4D).

To further test the role of the JAK/STAT pathway in mediating the stimulatory effect of YTHDF3 knockdown on PVSRIPO, we pretreated HeLa cells with IFN-α prior to infection and assessed the levels of viral translation upon YTHDF3 depletion (Fig. 4E). While YTHDF3 knockdown did not substantially alter viral translation in untreated HeLa cells (see also Fig. 2B), it significantly enhanced viral translation in IFN-α-pretreated HeLa cells. This corresponded with reduced levels of p-STAT1(Y701) and IFIT1 induction (Fig. 4E). Our findings suggested that YTHDF3 may play a role in regulating suppressor of cytokine signaling (SOCS) mRNA and protein expression, as the decay of mRNAs encoding these negative regulators of JAK/STAT signaling is regulated by m^6^A in T cells during murine development (33). However, in our system, transcripts encoding SOCS proteins or their functional homolog USP18 were not induced by YTHDF3 knockdown (Fig. S5A). Furthermore, SOCS1 and 3, which act specifically on type I-IFN-driven JAK/STAT1 signaling, remained unchanged at the protein level in YTHDF3-depleted cells before and during IFN-α2 treatment (Fig. S5B). Thus, the impact of YTHDF3 depletion on type I-IFN-driven JAK/STAT1 signaling occurs via a SOCS-independent mechanism.

10.1128/mBio.00116-21.6 FIG S5 The dampening of the ISG response upon YTHDF3 depletion is not due to induction of SOCS proteins. (A) A375 cells were transfected with ctrl siRNA or siRNA targeting YTHDF3 (see Table S1) 48 h prior to lysis for qRT-PCR analysis of the indicated transcripts. (B) The lysates from experiments reported in Fig. 4A and B (replicate 1) were run alongside an additional replicate 2 experiment to assess levels of SOCS1 and 3 proteins upon YTHDF3 depletion at baseline and after treatment with IFN-α (200 U/ml, 15 min). Immunoblots performed for YTHDF3, p-STAT1(Y701), and GAPDH in replicate 1 are shown in Fig. 4A (A375) and 4B (HeLa). Download FIG S5, TIF file, 1.2 MB. Copyright © 2021 Kastan et al. 2021 Kastan et al. https://creativecommons.org/licenses/by/4.0/ This content is distributed under the terms of the Creative Commons Attribution 4.0 International license.

## DISCUSSION

Proteolytic targeting of the YTHDF proteins by EV 2A^pro^ is consistent with recent reports that the cellular RNA methylation machinery, in the context of +ssRNA virus infection, is antiviral in nature (12). While genomic RNAs of EVs (13) and other +ssRNA viruses (12) were reported to be methylated, we focused on YTHDF proteins in the context of the host innate response to infection, as a large number of host cell transcripts (>7,000) are m^6^A-modified (11). Proteolytic degradation of YTHDF proteins in EV-infected cells occurs very early, prior to detectable viral translation, and before CPE manifests. Immediate early interference with YTHDF protein function implicates 2A^pro^-mediated cleavage as a viral ploy to oppose the antiviral host response, specifically induction of ISGs, in infected host cells. Indeed, preemptive YTHDF1, 2, or 3 depletion each enhanced viral translation and replication in PVSRIPO-infected cells that restrict early viral translation and eIF4G1 cleavage (A375, T3M4), resulting in inefficient viral 2A^pro^ activity and eIF4G1 cleavage. Confirming this role for viral 2A^pro^-mediated YTHDF1 to 3 cleavage, YTHDF3 depletion failed to stimulate viral translation in cells that intrinsically permit efficient viral translation and 2A^pro^-driven proteolytic events (HeLa, SUM149).

Deciphering the effects of YTHDF3 depletion on the innate host response to PVSRIPO infection indicated that the YTHDF proteins act at a step after IFN-β/IFN-λ1 release, implicating YTHDF3 as a mediator of JAK/STAT1 signaling. The classic EV-host cell interference event, 2A^pro^ activities associated with cleavage of eIF4G1/2, contributes to blocking host m^7^G-cap-dependent translation (18). A 2A^pro^-driven viral program to subvert host defenses in infected host cells may encompass concerted cleavages of YTHDF proteins and of eIF4G1/2, preventing ISG induction. A role for YTHDF proteins in facilitating the biosynthesis of critical innate antiviral proteins is compelling for several reasons. Cells harboring +ssRNA virus infection face acute inhibition of host protein synthesis, e.g., due to activation of the dsRNA-dependent protein kinase (PKR) or engaging of RNase L (34). Under these conditions of sudden-onset duress and global protein synthesis repression, cells must retain translation capacity for biosynthesis of critical host response factors such as ISGs. Alternatively, YTHDF3 may promote degradation of negative regulators of JAK/STAT1 signaling. This model is bolstered by a study showing that METTl3-deficient T cells are deficient in JAK/STAT5 signaling due to aberrant induction of multiple members of the suppressor of cytokine (SOCS) family of proteins, which negatively regulate this pathway (33). However, our investigations did not indicate a role of YTHDF3 in regulating SOCS/USP18 abundance at the transcriptional or translational levels.

A recent report suggested that YTHDF1 inhibits antigen cross-presentation in antigen-presenting cells (APC) via regulation of cathepsin expression and antigen degradation (35). PVSRIPO is in experimental use as a cancer immunotherapy agent in glioblastoma (36) and other indications. PVs naturally target APCs for infection (37), and PVSRIPO sublethal infection, lingering viral replication, and proinflammatory stimulation of tumor-associated APCs are implicated in instigating tumor antigen-specific antitumor immunity (38). Further studies must examine whether PVSRIPO 2A^pro^-mediated YTHDF cleavage occurs in this context *in vivo*, whether it can stimulate cross-presentation, and if it is involved in antitumor immunity elicited by PVSRIPO.

## MATERIALS AND METHODS

### Cell lines, *in vitro* transcription, and DNA/RNA transfections.

HeLa R19 (39), SUM149 (40), A375, and T3M4 cells (both ATCC) were grown in Dulbecco’s modified Eagle’s medium (DMEM) supplemented with 10% fetal bovine serum (FBS) and nonessential amino acids. siRNA transfections were performed 36 h prior to infection at ∼50% confluence with 50 pM siRNA and 5 μl of Lipofectamine RNAiMax (Invitrogen) per well (6-well plate) in serum-free media. The sequences of siRNAs employed in this study are shown in Table S1 in the supplemental information. Myc-/Flag-tagged YTHDF3 was purchased from Origene. The Y90A/G91A mutations eliminating the 2A^pro^ cleavage site were introduced by QuikChange lightning site directed mutagenesis kit (Agilent) using primer pair 5′-ATGTTCTCCATTACTCATTTGTGCAGCGGTTGTCAGATATGGCATAGGC-3′/5′-GCCTATGCCATATCTGACAACCGCTGCACAAATGAGTAATGGAGAACAT-3′. These constructs were then transfected (6 h) with Lipofectamine 2000 (Invitrogen) into cells at 90% confluence according to the manufacturer’s protocol, 24 h prior to infection. WT and C109A 2A^pro^ RNA constructs have been described previously (28). These constructs were *in vitro* transcribed using the mMESSAGE mMACHINE T7 transcription kit (Invitrogen). This capped RNA was then transfected (3 μg; 12 h) with DMRIE-C (Invitrogen) according to the manufacturer’s protocol.

### Viral infections, inhibitors, cytokines, and one-step growth curves.

PVSRIPO and CBV3 infections were performed at an MOI of 10 in DMEM supplemented with 1% FBS and nonessential amino acids (39, 41). Z-VAD-FMK and Ruxolitinib (Tocris) were dissolved in DMSO and used at the indicated concentrations. IFN-γ and IFN-α2 (PBL) were used at the indicated concentrations. One-step growth curves and plaque assays were performed as reported before (39, 41).

### Immunoprecipitation, immunoblotting, RT-qPCR, and LEGENDplex cytokine analysis.

Lysate preparation, immunoprecipitation (IP), and immunoblotting have been described previously (42). For IPs, cells were lysed in radioimmunoprecipitation assay (RIPA) buffer (Millipore) with halt protease-phosphatase inhibitor cocktail (Thermo Scientific). After overnight incubation with anti-Flag beads (Thermo Scientific), beads were washed 4 times in RIPA buffer and processed for immunoblotting. Total protein stains were performed using total protein stain (Li-COR). Quantifications for immunoblot were normalized to total protein. Blots were developed using Western Bright (BioExpress) or SuperSignal West Pico/Femto (Thermo Scientific) chemiluminescence (ECL) kits. Antibodies against YTHDF1, YTHDF2, YTHDF3 (Proteintech), eIF4G1, G3BP1, PABP, GAPDH, p-STAT1(Y701), p-IRF3(S396), IRF3, STAT1, MDA5, IFIT1, ISG15 (Cell Signaling Technology), Flag (Invitrogen), poliovirus 2C (42), and CVB3 3D (a generous gift from K. Klingel, University of Tübingen) were used. For reverse transcriptase quantitative PCR (RT-qPCR), cells were lysed directly in TRIzol (Invitrogen) to isolate total RNA. The samples were treated with DNase (NEB) prior to cDNA synthesis (Invitrogen SuperScript III) following the manufacturer’s protocol. RT-qPCR was performed using SYBR green (Thermo) on a QuantStudio3 machine (Thermo). The sequences of primers used for RT-qPCR analyses in this study are shown in Table S1. For cytokine release assays, the LEGENDplex human antiviral kit was used as described before (29).

### Statistical analysis.

Quantification of immunoblots was performed with the Li-COR Odyssey FC2 imaging system and Image Studio software. All experiments were repeated at least 3 times. Normalization methods for quantified immunoblot data are described in the figure legends and were represented as averages and standard errors of the means (SEM). The two-tailed Student’s *t* test was used for each comparison. Significance was defined as a *P* value of <0.05 and described in figure legends.
