# Supplementary figures and images for: Enterovirus 2Apro Cleavage of the YTHDF m6A Readers Implicates YTHDF3 as a Mediator of Type I Interferon-Driven JAK/STAT Signaling
Source: mBio. 2021 Apr 13;12(2):e00116-21. doi: 10.1128/mBio.00116-21 (PMC8092205; doi:10.1128/mBio.00116-21)

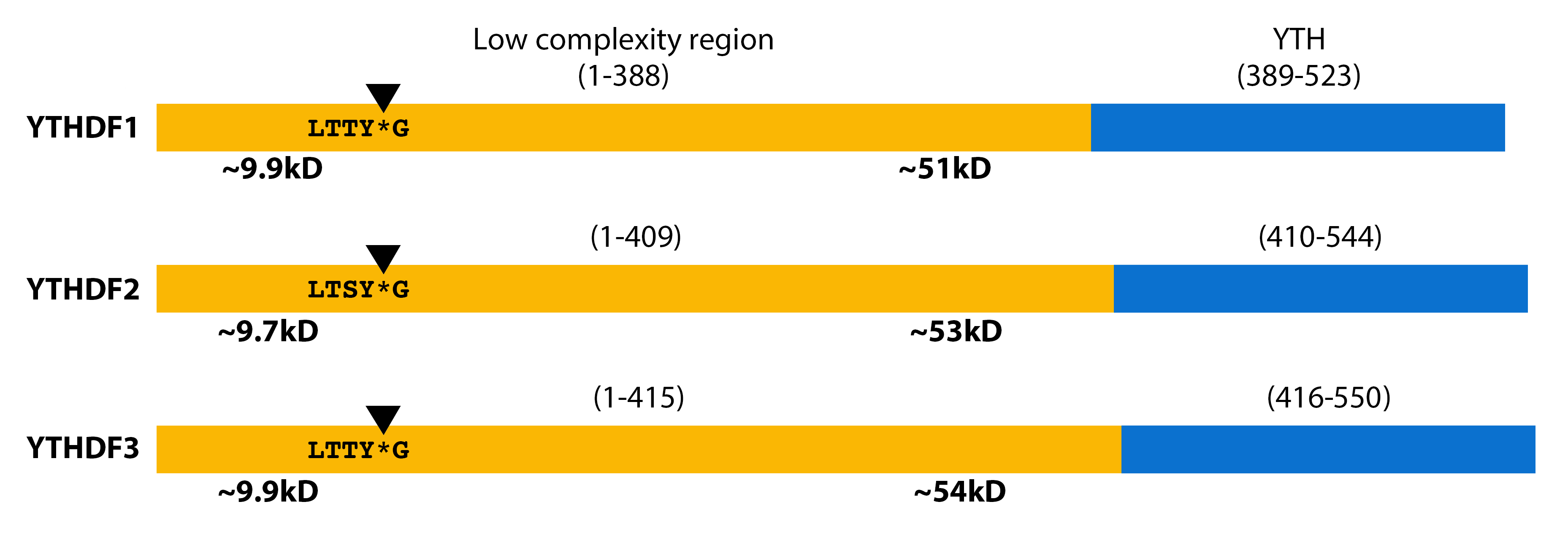

Supplement: FIG S1 [file mBio.00116-21-sf001.tif]

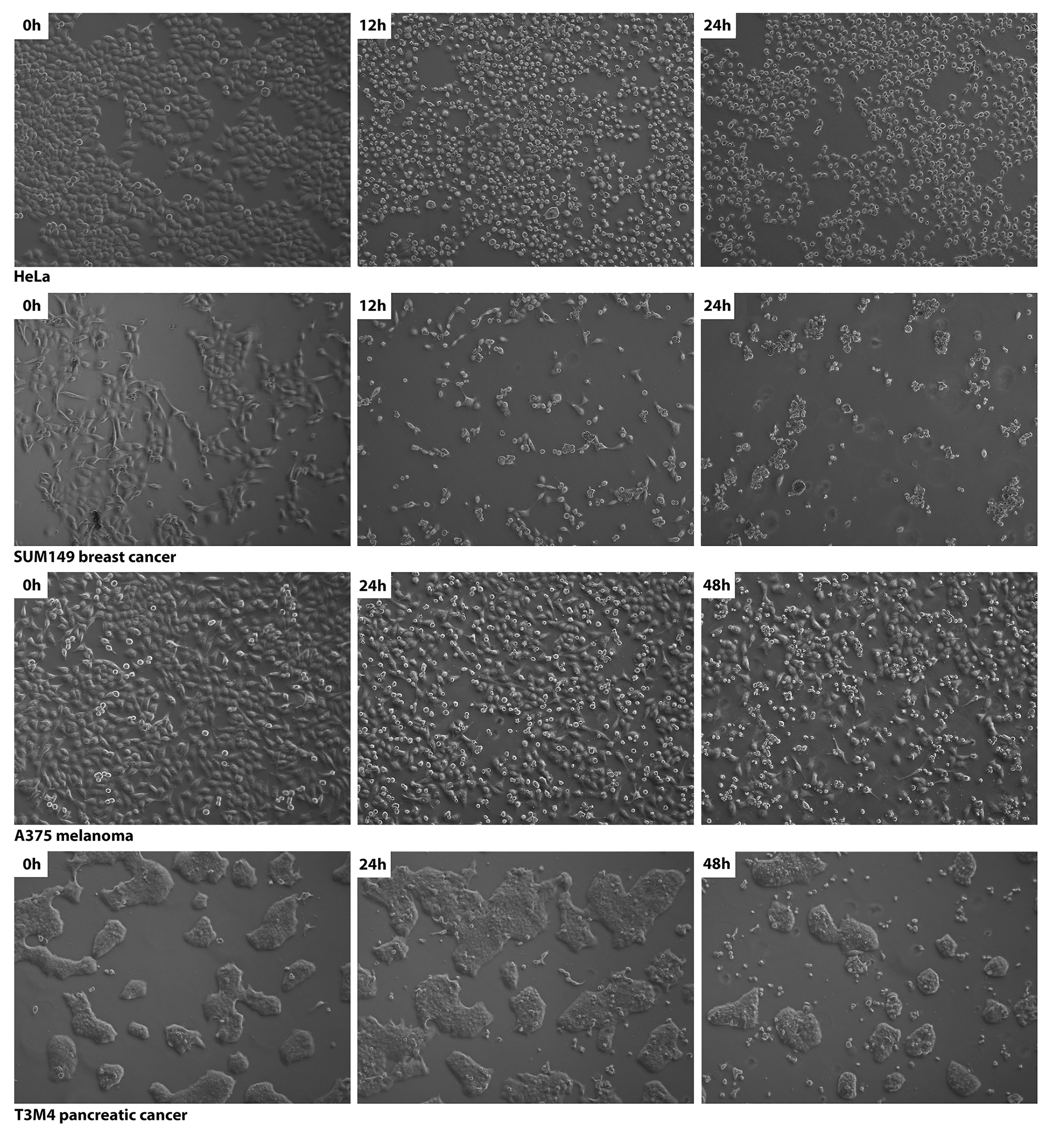

Supplement: FIG S2 [file mBio.00116-21-sf002.tif]

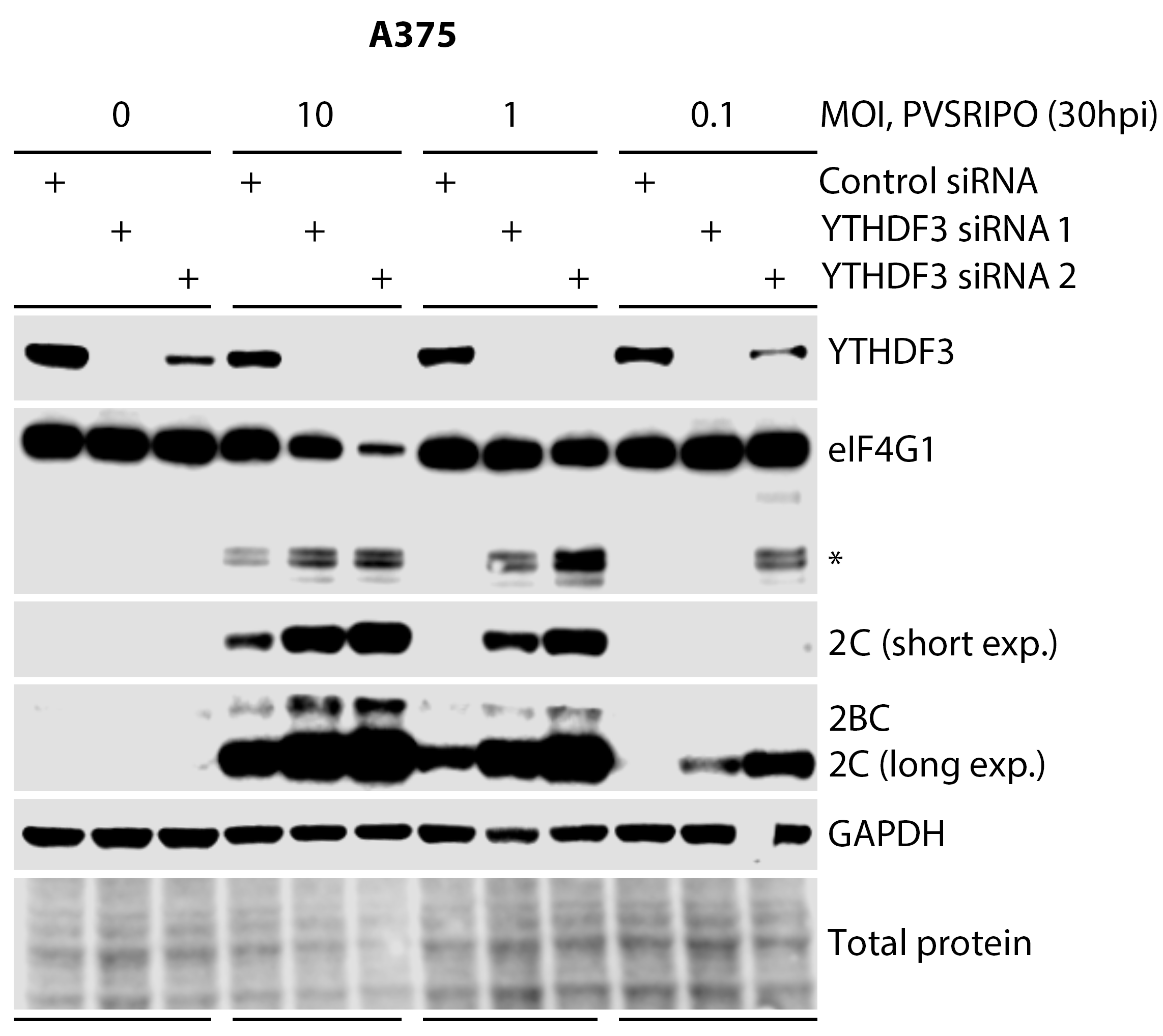

Supplement: FIG S3 [file mBio.00116-21-sf003.tif]

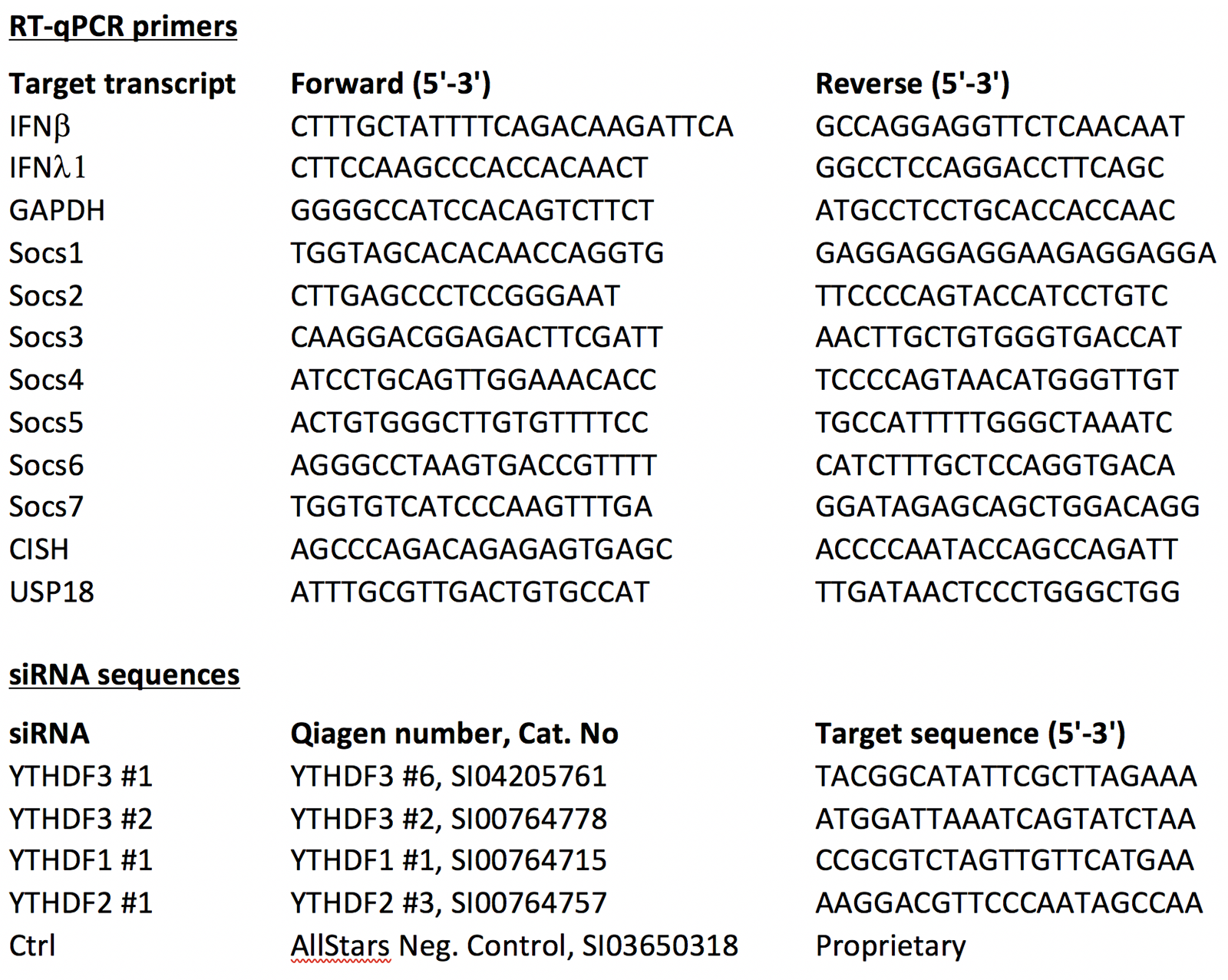

Supplement: TABLE S1 [file mBio.00116-21-st001.tif]

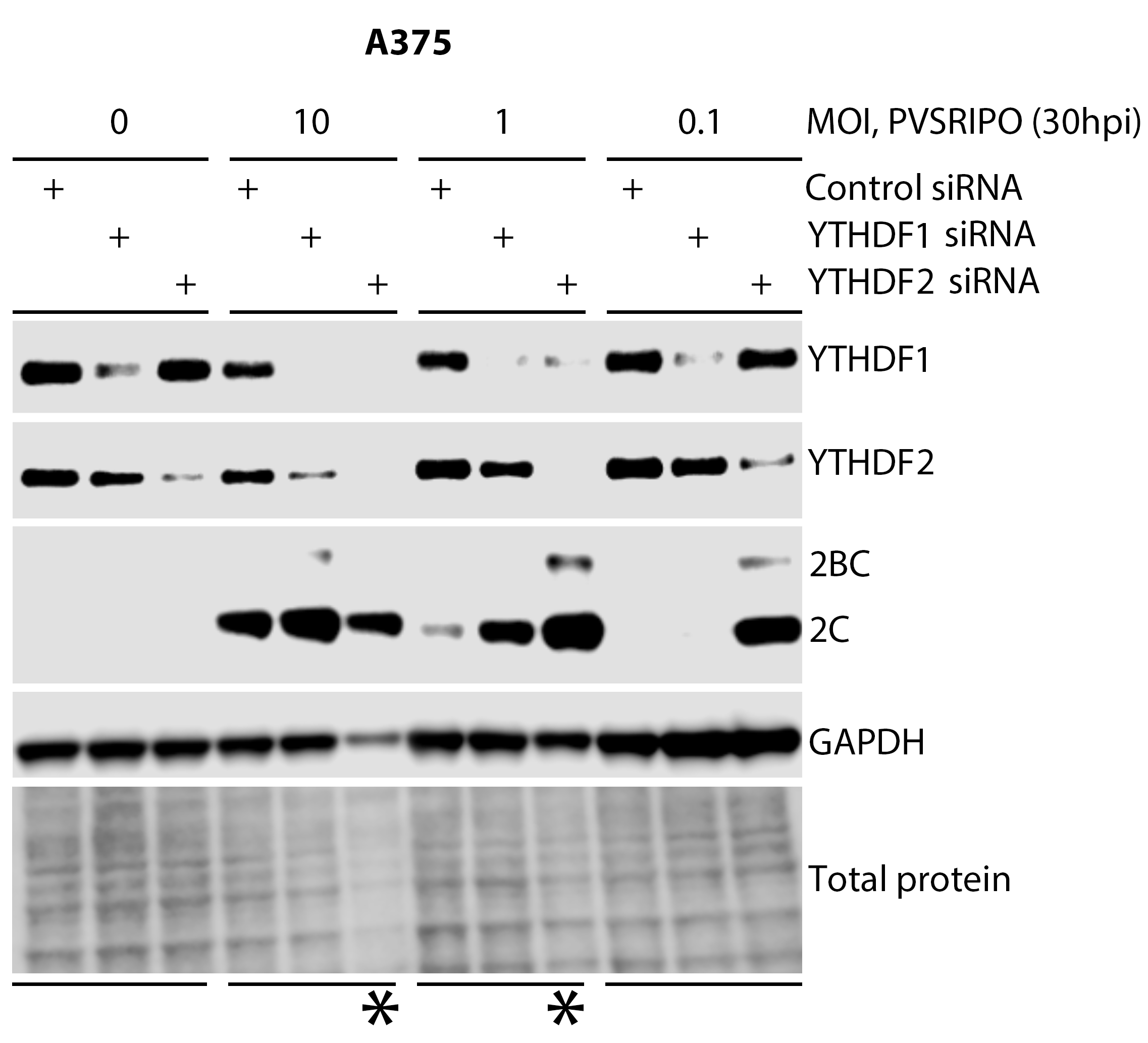

Supplement: FIG S4 [file mBio.00116-21-sf004.tif]

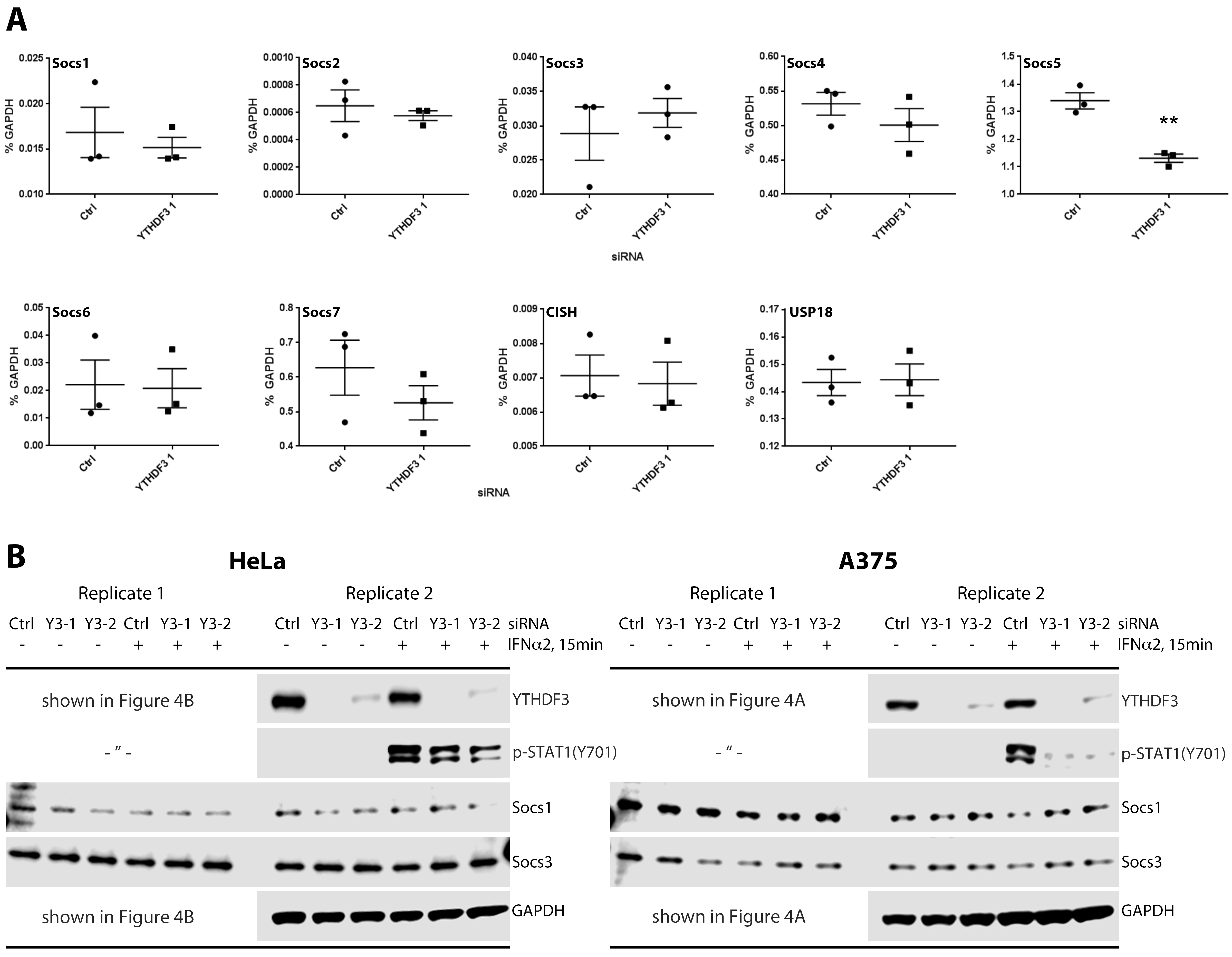

Supplement: FIG S5 [file mBio.00116-21-sf005.tif]
